# Salumycin, a New Pyrazolequinone from a *Streptomyces albus* J1074 Mutant Strain

**DOI:** 10.3390/molecules25184098

**Published:** 2020-09-08

**Authors:** Kaixiang Tao, Taijia Ye, Mingming Cao, Xiaolu Meng, Yuqing Li, Huan Wang, Zhiyang Feng

**Affiliations:** 1 College of Food Science and Technology, Nanjing Agricultural University, 1 Weigang, Nanjing 210095, China; 2017108011@njau.edu.cn (K.T.); 2017108012@njau.edu.cn (T.Y.); caomingming@njau.edu.cn (M.C.); 2015108019@njau.edu.cn (X.M.); 2 School of Chemistry and Chemical Engineering, Nanjing University, 163 Xianlin Avenue, Nanjing 210023, China; lyqdzx@126.com

**Keywords:** pyrazolequinone, *Streptomyces albus*, biosynthetic gene activation, antioxidant activity

## Abstract

Heterocyclic natural products with various bioactivities play significant roles in pharmaceuticals. Here, we isolated a heterocyclic compound salumycin (**1**) from a *Streptomyces albus* J1074 mutant strain. The structure of (**1**) was elucidated via single-crystal X-ray diffraction, mass spectrometry (MS), fourier transform infrared spectrometer (FTIR), and nuclear magnetic resonance (NMR) data analysis. Salumycin (**1**) contained a novel pyrazolequinone ring, which had never been previously reported in a natural product. Salumycin (**1**) exhibited moderate 2,2′-diphenyl-1-picrylhydrazyl (DPPH)-radical scavenging activity (EC50 = 46.3 ± 2.2 μM) compared with *tert*-butylhydroquinone (EC50 = 4.7 ± 0.3 μM). This study provides a new example of discovering novel natural products from bacteria.

## 1. Introduction

Bacteria are an important source of novel natural products and many bacterial natural products or their derivatives are currently used in human and animal pharmaceuticals, the food industry, and crop protection [1,2]. In recent years, the discovery of natural products from bacterial sources has shown an increasingly high repetition rate [3]. In laboratory conditions, most of the environmental bacteria cannot be cultured [4], and the majority of natural product biosynthetic gene clusters on bacterial genomes are silent [5]. Currently, a metagenomic approach has been applied for the discovery of novel natural products from uncultured bacteria [6], and methods including the change in growth conditions [7], manipulation of the pathway-specific regulators [8], clustered regularly interspersed short palindromic repeats (CRISPR)-Cas9 strategy [9], and heterologous expression [10] have been used to activate the silent biosynthetic gene clusters. Heterologous expression is widely used as a strategy for the activation of cryptic natural product biosynthetic gene clusters and the improvement of natural product yield [11].

Heterocyclic natural products are unique compounds that have a wide range of biological, chemical, and physical characteristics, and more than half of small-molecule drugs contain a heterocyclic moiety, frequent examples are indole, pyridine, and thiazole as well as quinazoline rings [12,13].

In our previous study, we constructed a metagenomic library derived from the environmental bacterial genomic DNA [14]. We then screened clones harboring putative natural product biosynthetic genes from the metagenomic library and transferred the positive clones into *Streptomyces albus* J1074 for the discovery of new natural products. A *S. albus* mutant that could produce a new pyrazolequinone compound was obtained during the conjugation. Herein, identification of the *S. albus* mutant as well as the isolation, structure determination, and antioxidant activity of the new pyrazolequinone compound are reported.

## 2. Results and Discussion

**Figure 1:**
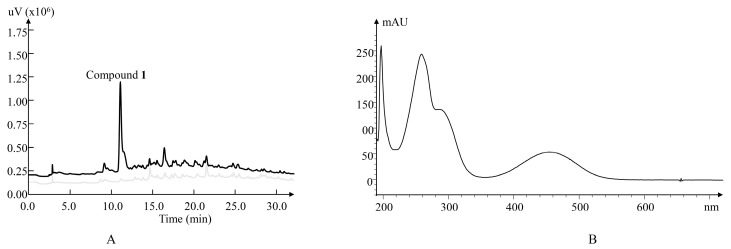
(**A**). HPLC profile of crude extract of the *S. albus* J1074 mutant (black) and wild-type (gray) strain. (**B**). UV-Vis spectrum of (**1**) in MeOH.

To obtain positive clones harboring natural product biosynthetic genes for new compound discovery, we screened the library using a pair of primers derived from the conserved sequence of halogen genes and identified positive clones. We then transferred a clone into *Streptomyces albus* J1074 by conjugation [15]. A red conjugant was identified, which showed a different phenotype from other white conjugants on the conjugation plate. The red clone was analyzed first by polymerase chain reaction (PCR) using halogen primers (Figure S2). The halogen gene fragment could not be amplified by PCR and the following genome sequencing showed that there was a big deletion in the mutant genome (from 6,356,863 bp to 6,841,649 bp) and both the *attB* and the pseudo-*attB* sites were occupied by part of the conjugation vectors. This mutated strain was fermented and the ethyl acetate extract of the fermentation broth was analyzed by high performance liquid chromatography (HPLC). A peak was identified in the HPLC trace (Figure 1A), and its UV-Vis spectrum (Figure 1B) was different from other known compounds derived from *S. albus* J1074. To investigate this compound, we accumulated it from the ethyl acetate extract of 10 L fermentation cultures of the mutant. The isolation and characterization of the compound revealed a new pyrazolequinone salumycin (**1**).

**Figure 2:**
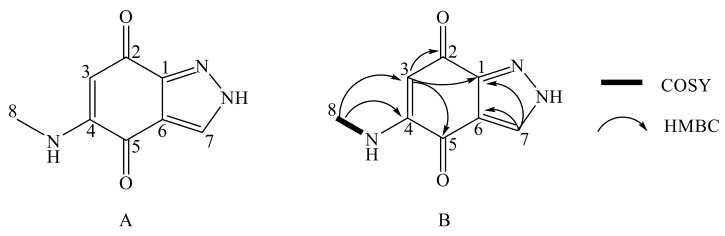
(**A**). Chemical structure of **1**. (**B**). Key 2D NMR correlations of **1**.

Salumycin (**1**) was isolated as a red powder. Its molecular formula was established as C_8_H_7_N_3_O_2_ (*m*/*z* 178.0615, [M + H]^+^, calcd. for 178.0616) with seven degrees of unsaturation by high-resolution electrospray ionization mass spectroscopy (HRESIMS). The infrared (IR) spectrum indicated the presence of the methyl group (2874 and 2961 cm^−1^), the carbonyl group (1585 cm^−1^), the amino group (3342 cm^−1^), the pyrazole moiety, and benzene ring (3084, 1515, 1489, 1455, 1406, 1242, and 790 cm^−1^) (Figure S3). The UV-Vis absorption (Figure 1B) at 455 nm indicated a conjugated system. The ^1^H NMR spectrum of **1** (Table 1) revealed five signals, corresponding to a doublet methyl signal (*δ*_H_ 2.76), two olefinic proton signals (*δ*_H_ 5.28 and 8.30), and two amino proton signals (*δ*_H_ 7.68 and 14.19). The ^13^C NMR and DEPT 135 spectra (Figure S6) of 1 revealed the presence of eight carbons including a methyl (*δ*_C_ 29.3), two olefinic methines (*δ*_C_ 97.6 and 133.4), and five quaternary carbons (*δ*_C_ 117.4, 146.1, 151.5, 176.7, and 176.9). The ^1^H-^1^H COSY correlations established the presence of a methylamino group (CH_3_-NH-) between H_3_-8 (*δ*_H_ 2.76) and 4-NH (*δ*_H_ 7.68) (Figure 2B). The methyl protons H_3_-8 (*δ*_H_ 2.76) indicated significant heteronuclear multiple-bond correlation (HMBC) correlations with C-3 (*δ*_C_ 97.6), C-4 (*δ*_C_ 151.5), confirming the attachment of a methylamino group (CH_3_-NH-) to C-4 (Figure 2B). Furthermore, two olefinic methine protons (H-3 and H-7) from **1** showed HMBC correlations from H-3 to C-1, and H-7 to C-1/C-6. Since the ^13^C chemical shifts of C-2 (*δ*_C_ 176.7/176.9) and C-5 (*δ*_C_ 176.7/176.9) were very close, the HMBC correlations from H-3 to C-2 and C-5 were not distinct (Figure S9).

**Figure 3:**
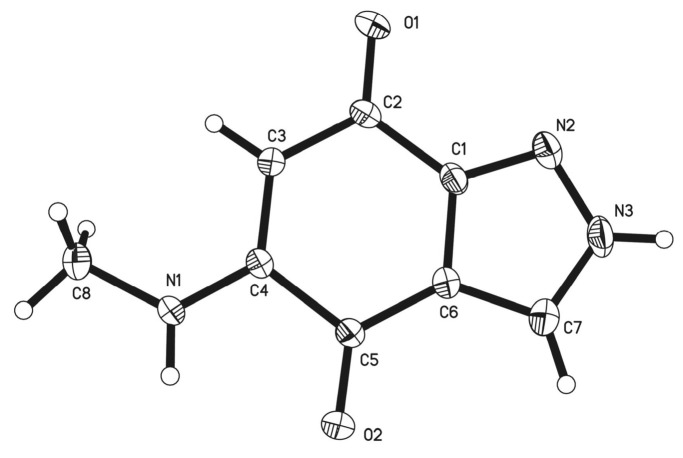
X-ray crystal structure of **1**.

Since the NMR analysis was not able to fully assign the structure of salumycin (**1**) (Figure 2B), we obtained the crystal of salumycin (**1**) in acetone solvent at room temperature. The structure of (**1**) was then unambiguously determined by X-ray single-crystal diffraction analysis (Figure 2A, Figure 3) (CCDC 1973192). Accordingly, the big deletion of the genome and/or the insertion in the *attB* and pseudo-*attB* sites of the mutant strain might activate the silent biosynthetic genes on the genome of *S. albus* J1074, leading to the production of (**1**). Recently, indazole-4,7-dione derivatives that are structurally similar to (**1**) have been chemically synthesized and their biological activity of inhibiting protein BRD4, which could control the proliferation of cancer cells, was reported [16]. Here, we obtained (**1**) as a natural product from a *S. albus* J1074 mutant strain.

The antioxidant activity of (**1**) was evaluated by the DPPH free radical scavenging assay [17]. Compared with the high DPPH free radical scavenging activity of *tert*-butylhydroquinone (EC50 = 4.7 ± 0.3 μM), (**1**) showed moderate activity (EC50 = 46.3 ± 2.2 μM). Meantime, the 2,2′-azino-bis(3-ethylbenzothiazoline-6-sulfonic acid) diammonium salt (ABTS), hydroxyl, and superoxide anion radical scavenging assay of (**1**) were measured, and no activity was detected (data not shown). We also tested the antibacterial activity of (**1**) and almost no antibacterial activity was detected. In addition, we predicted the bioactivity of (**1**) with molinspiration software. The bioactivity scores of (**1**) as the G protein-coupled receptor (GPCR) ligand (−0.61), ion channel modulator (−0.53), kinase inhibitor (0.31), nuclear receptor ligand (−1.13), protease inhibitor (−1.04), and enzyme inhibitor (0.27) suggested (**1**) has the potential to act as kinase and enzyme inhibitor.

Although *S. albus* J1074 has widely been used as a heterologous expression strain for natural product discovery [18], *S. albus* J1074 could produce novel compounds by activating the natural product biosynthetic gene clusters on its genome. Five groups of natural products have been isolated by genome-mining and over-expression of positive regulators to activate silent gene clusters on the genome of *S. albus* J1074 [19]. Recently, a novel isoindolequinone compound albumycin was identified by a ‘cross-talk’ between a heterologous gene cluster and a native gene cluster in *S. albus* J1074 [20]. In this study, we found a novel pyrazolequinone compound by activating a silent gene cluster in the genome of *S. albus* J1074. The genome sequencing showed that there was a big deletion in the genome DNA and both *attB* and pseudo-*attB* sites were occupied by conjugation vectors, thus, the gene cluster of (**1**) might be activated by a different way from that of albumycin. Moreover, only salumycin (**1**) was identified from the mutated strain in this study, and (**1**) contained a novel pyrazolequinone ring system, which has never been previously reported as a natural product. Thus, (**1**) and albumycin might be derived from different gene clusters in *S. albus* J1074 that have never been reported before. Nonetheless, further research is required to reveal the biosynthetic machinery of (**1**) and how it is activated.

## 3. Materials and Methods

### 3.1. General Experimental Procedures

UV spectrum was measured with a UV-2600 (Unico, Shanghai, China). 1D and 2D NMR spectra were recorded on the Avance-500 MHz spectrometer (Bruker, Karlsruhe, Germany). HRESIMS was measured with a Triple TOF 4600(AB SCIEX, Framingham, MA, USA). IR spectrum was recorded on Nexus-870 Fourier transform infrared spectrometer (Thermo Necolet, Waltham, MA, USA). X-ray crystallographic data were collected on a SMART APEX-Ⅱ (Bruker-AXS, Billerica, MA, USA). Column chromatography was performed on silica gel (200–300 mesh, Qingdao Marine Chemicals, Qingdao, China). Analytical reversed phase HPLC was performed on a SHIMADZU LC-20A with a InertSustain C18 column (250 × 4.6 mm, 5 μm, SHIMDAZU, Kyoto, Japan), and semi-preparative HPLC was performed on the same instrument with a SHIMDAZU Shim-pack GIS-C18 column (250 mm × 10 mm, 10 μm, SHIMDAZU, Kyoto, Japan).

### 3.2. Bacterial Strains, Plasmid, and Media

*Streptomyces albus* J1074 was used as the host strain for heterologous expression. *E. coli* EPI100 (Epicentre, Madison, WI, USA) and pWEB-TNC (Epicentre, Madison, WI, USA) were used as the host and vector for library construction, respectively. *E. coli* ET12567 (Biovector NTCC Inc., Beijing, China) and pOJ436 (Biovector NTCC Inc., Beijing, China) were used as the donor strain and retrofitting vector for conjugation, respectively. Luria-bertani (LB) medium (5 g yeast extract (Sangon Biotech, Shanghai, China), 10 g tryptone (Sangon Biotech, Shanghai, China), 10 g NaCl (Sangon Biotech, Shanghai, China) in 1 L water) was used for culturing *E. coli* strains. Tryptic soy broth (TSB) medium (17 g tryptone, 3 g soy peptone (Sangon Biotech, Shanghai, China), 2.5 g glucose (Sangon Biotech, Shanghai, China), 5 g NaCl, 2.5 g K_2_HPO_4_ (Nan jing Reagent Co., Ltd, Nanjing, China) in 1 L water) was used for culturing the *Streptomyces* strain to prepare genome DNA. R5 medium (103 g sucrose (Sangon Biotech, Shanghai, China), 0.25 g K_2_SO_4_ (Sinopharm Chemical Reagent Co., Ltd, Shanghai, China), 10.12 g MgCl_2_·6H_2_O (Macklin, Shanghai, China), 10 g glucose, 0.1 g casein amino acid, 2 mL trace element solution, 5 g yeast extract, 5.73 g *N*-tris-(hydroxymethyl)methyl-2-aminoethane sulfonic acid (TES) (Sangon Biotech, Shanghai, China) in 1 L water) was used as the seed medium, and R5 medium containing 5% HP-20 resin (Generay Biotechnology, Shanghai, China) was used as the production medium during the fermentation of *Streptomyces*.

### 3.3. PCR-Based Screening for Clones Containing Halogen Genes

A pair of primers (Trp-FW: 5′-TCGGSGTSGGCGARGCSACCKT-3′, Trp-RV: 5′-CGGTRSWCTCCAGCGGCTCGACGAA-3′) [21], which were designed based on the conserved sequences of the FADH_2_-dependent tryptophan halogenase genes, were used to screen the Tibet soil metagenomic library [14] by PCR (95 °C for 3 min, followed by 35 cycles of 94 °C for 45 s, 64 °C for 40 s, 72 °C for 75 s, and finally 72 °C for 10 min). Amplicons with the correct predicted size (750–900 bp) were gel-purified and sequenced. The sequences were then analyzed by basic local alignment search tool (BLAST, Bethesda, MD, USA, https://blast.ncbi.nlm.nih.gov/) to identify the halogen genes. The halogen-gene-containing clones were then obtained by the serial dilution method [22] from the library (constructed by ourselves).

### 3.4. Identification of S. albus J1074 Mutant Strain

The positive clones were retrofitted with pOJ436 and transferred into *E. coli* ET12567, then transferred into *S. albus* J1074 by conjugation [15]. Conjugates could be seen on MS medium after 3 d and one red clone, which was different from other conjugates on the same plate, was identified. The genome DNA of this red clone was extracted following the protocol described [23], and used as the template for PCR using Trp-FW/Trp-RV primers. The whole genome of the red clone was also sequenced by the Nanopore sequencing method [24] as following: first, the quality of the genomic DNA was checked for conformity (appearance, purity, size, and quantification). Then, a DNA library was constructed. After quantitative detection, the DNA library of a certain concentration and volume was added to a flow cell and transferred the flow cell to the PromethION sequencer for real-time single molecule sequencing. This sample sequenced a cell using the PromethION sequencing platform, which produced a total of 7,492,414,971 bp raw data with a reads number of 760,966, reads mean length of 9845 bp, N50 reads of 13,334 bp, and longest reads of 164,396 bp. The amount of data through quality control was 7,462,705,631 bp. After the quality control, data were assembled, corrected, and optimized, and the final genome data were obtained. The data contained one contig with a size of 6,375,258 bp, GC content of 73.18%, and sequencing depth of 1070.96 X. After sequence analyzing, there was a big deletion in the *S. albus* J1074 genome (from 6,356,863 bp to 6,841,649 bp) and both the *attB* and the pseudo-*attB* sites were occupied by the conjugation vectors. The genomic sequencing was completed by Nextomics Biosciences Co. Ltd. (Wuhan, China).

### 3.5. Fermentation and Isolation of Salumycin (***1***)

Ten μL of spore of the red strain was inoculated into 40 mL of seed medium in a 250 mL flask and cultured at 28 °C on a rotary shaker (225 rpm) for 2–3 days. Then, 6 mL of the culture was inoculated into 40 mL of production medium in 250 mL flask. The fermentation was performed at 28 °C for 13 days on a rotary shaker (225 rpm). After the fermentation, the resin in the production medium was collected and washed with methanol to extract the metabolites. The metabolites were analyzed by HPLC (1 mL/min) using a linear gradient of 80:20 H_2_O:MeOH to 100% MeOH over 30 min. For big scale fermentation, 10 L production medium was inoculated and 7.6 g of crude extract was finally obtained. The crude extract was initially fractioned by silica gel (200–300 mesh) flash chromatography using a CH_2_Cl_2_:MeOH step gradient. Salumycin (**1**) was eluted from this column with 100:3 and 100:4 CH_2_Cl_2_:MeOH, and was purified by semi-preparative HPLC (3ml/min) using a linear gradient of 80:20 to 20:80 of H2O:MeOH over 50 min. Finally, 5.2 mg of salumycin (**1**) was obtained (*t*_R_ = 11.1 min).

### 3.6. 2,2′-Diphenyl-1-Picrylhydrazyl (DPPH) Free Radical Scavenging Assay

The assay was performed as described by Gao et al. [17]. The compounds were dissolved in MeOH to form a concentration gradient of 3.125–100 μg/mL. DPPH was dissolved in MeOH to form a concentration of 0.1 mM. The mixture (compound and DPPH) was placed in a 96-well plate, kept in the dark for 30 min, and its absorbance was measured with a microplate reader (Synergy 2, Bio Tek) at 517 nm. The DPPH radical scavenging rate of samples was calculated as follows:

Scavenging rate (%) = [1–{(A_1_ − A_2_)/A_0_}] × 100

A_0_ is the absorbance of the negative control (DPPH), A_1_ is the absorbance of the mixture (compound and DPPH), and A_2_ is the absorbance of the compound dissolved in MeOH.

### 3.7. In Silico Bioactivity Assay

The bioactivity scores of (**1**) as the GPCR ligand, ion channel modulator, kinase inhibitor, nuclear receptor ligand, protease inhibitor, and enzyme inhibitor were calculated by using molinspiration online (Bratislava, Slovak Republic, https://www.molinspiration.com/), according to the standard structure of the compound. The scores of more than 0.00, −0.50 to 0.00, and less than −0.50 corresponded to active, moderately active, and inactive bioactivities, respectively.

### 3.8. Crystal Data

Crystal data for C_8_H_7_N_3_O_2_ (M = 177.17 g/mol): monoclinic, space group P2**_1_**/c (no. 14), a = 4.8856 (3) Å, b = 10.0508 (7) Å, c = 16.1190 (12) Å, β = 95.743 (2)°, V = 787.54 (9) Å^3^, Z = 4, T = 296 (2) K, μ(MoKα) = 0.112 mm^−1^, Dcalc = 1.494 Mg/m^3^, 8627 reflections measured (5.08° ≤ 2θ ≤ 54.856°), 1786 unique (Rint = 0.0257, Rsigma = 0.0229), which were used in all calculations. The final R1 was 0.0422 (I > 2σ (I)) and wR2 was 0.1120.

## 4. Conclusions

In summary, a new compound salumycin (**1**) was isolated from a *S. albus* J1074 mutant. Salumycin (**1**) contains a novel pyrazolequinone ring that has never been reported before in natural products. Salumycin (**1**) exhibited DPPH-radical scavenging activity, but no antibacterial activity. Genome analysis of the mutant showed a big genome deletion and exogenous DNA insertions. The biosynthetic mechanism of (**1**) needs to be further investigated and this study highlights again that bacteria are still an important resource for the discovery of novel natural products.

## Figures and Tables

**Table 1:** ^1^H (500 MHz) and ^13^C (125 MHz) NMR data of (**1**) in dimethyl sulfoxide (DMSO)-*d*_6_.

NO.	*δ*_C_, Type	*δ*_H_, *J* in Hz
1	146.1, C	
2	176.7/176.9, C	
3	97.6, CH	5.28, s
4	151.5, C	
5	176.9/176.7, C	
6	117.4, C	
7	133.4, CH	8.30, s
8	29.3, CH3	2.76, d, 5.2
4-NH		7.68, d, 5.7
NH-7		14.19, br s

## Supplementary Materials

The following are available online at https://www.mdpi.com/1420-3049/25/18/4098/s1. Figure S1: HRESIMS spectrum of (**1**), Figure S2: PCR verification of the genome of the red clone, Figure S3: FTIR-spectrum of (**1**), Figure S4: DPPH free radical scavenging activity of (**1**), Figure S5: ^1^H NMR spectrum of (**1**) in DMSO-*d*6, Figure S6: DEPT 135 and^13^C NMR spectrum of (**1**) in DMSO-*d*6, Figure S7: HSQC spectrum of (**1**) in DMSO-*d*6, Figure S8: COSY spectrum of (**1**) in DMSO-*d*6, Figure S9: HMBC spectrum of (**1**) in DMSO-*d*6, Table S1: Crystal data and structure refinement of (**1**), Table S2: Bond lengths and angels of (**1**), Table S3: Hydrogen bonds of (**1**).
