# Supplementary material for: Salumycin, a New Pyrazolequinone from a Streptomyces albus J1074 Mutant Strain
Source: Molecules. 2020 Sep 8;25(18):4098. doi: 10.3390/molecules25184098 (PMC7570766; doi:10.3390/molecules25184098)
Supplement: Supplementary file 1 [file molecules-25-04098-s001.pdf]

molecules

SUPPLEMENTARY MATERIAL

Salumycin, a New Pyrazolequinone from a *Streptomyces albus* J1074 Mutant Strain

Kaixiang Tao [1], Taijia Ye [1], Mingming Cao [1], Xiaolu Meng [1], Yuqing Li [2], Huan Wang [2,*] and Zhiyang Feng [1,*]

[1] College of Food Science and Technology, Nanjing Agricultural University, 1 Weigang, Nanjing 210095, China; 2017108011@njau.edu.cn (K.T.); 2017108012@njau.edu.cn (T.Y.); caomingming@njau.edu.cn (M.C.); 2015108019@njau.edu.cn (X.M.)

[2] School of Chemistry and Chemical Engineering, Nanjing University, 163 Xianlin Avenue, Nanjing 210023, China; lyqdzx@126.com

* Correspondence: wanghuan@nju.edu.cn (H.W.); zfeng@njau.edu.cn (Z.F.);
Tel./Fax: +86-025-89682133 (H.W.); +86-025- 84399511 (Z.F.);

Table of contents

Figure S1. HRESIMS spectrum of (**1**) 3
Figure S2. PCR verification of the genome of the red clone........ 4
Figure S3. FTIR-spectrum of (**1**) 5
Figure S4. DPPH free radical scavenging activity of (**1**) 6
Figure S5. ^{1}H NMR spectrum of (**1**) in DMSO-*d*6 7
Figure S6. DEPT 135 and^{13}C NMR spectrum of (**1**) in DMSO-*d*6........ 8
Figure S7. HSQC spectrum of (**1**) in DMSO-*d*6........ 9
Figure S8. COSY spectrum of (**1**) in DMSO-*d*6 10
Figure S9. HMBC spectrum of (**1**) in DMSO-*d*6........11
Table S1. Crystal data and structure refinement of (**1**) 12
Table S2. Bond lengths and angels of (**1**) 13
Table S3. Hydrogen bonds of (**1**)........ 14

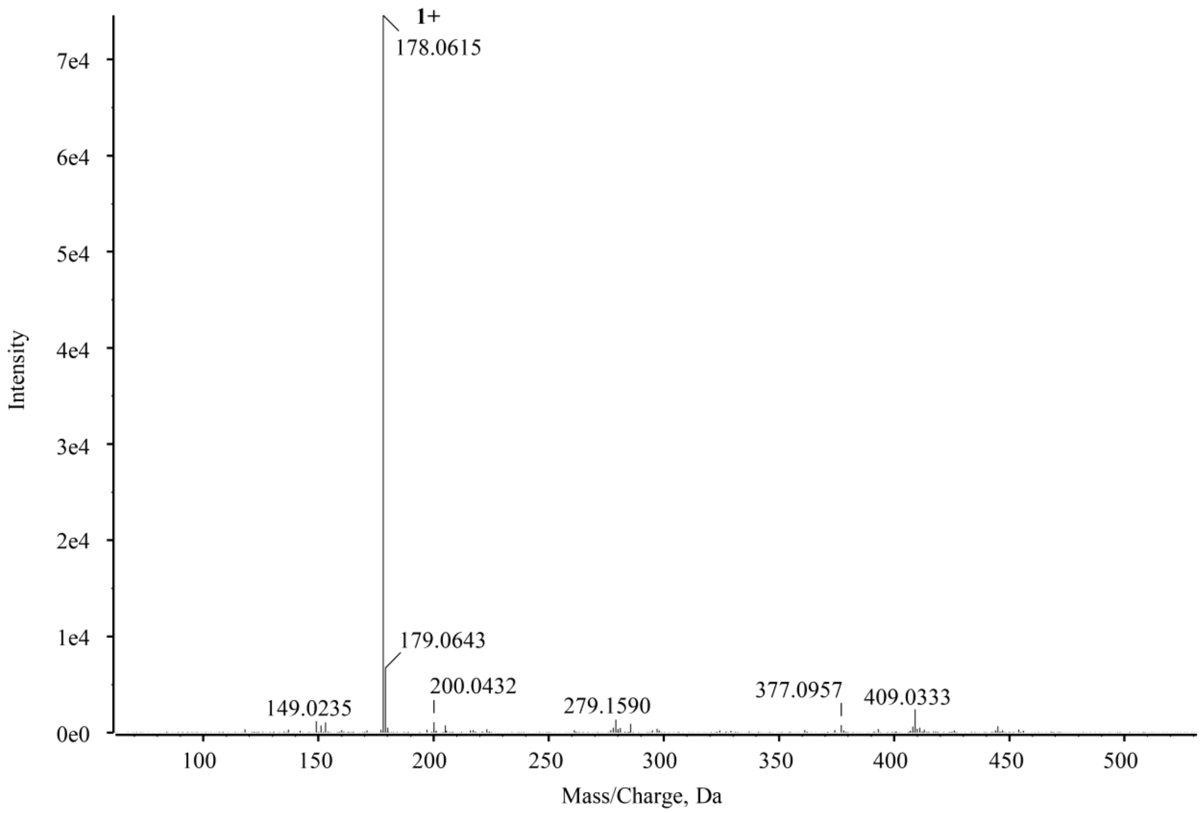

Figure S1. HRESIMS spectrum of **(1)**

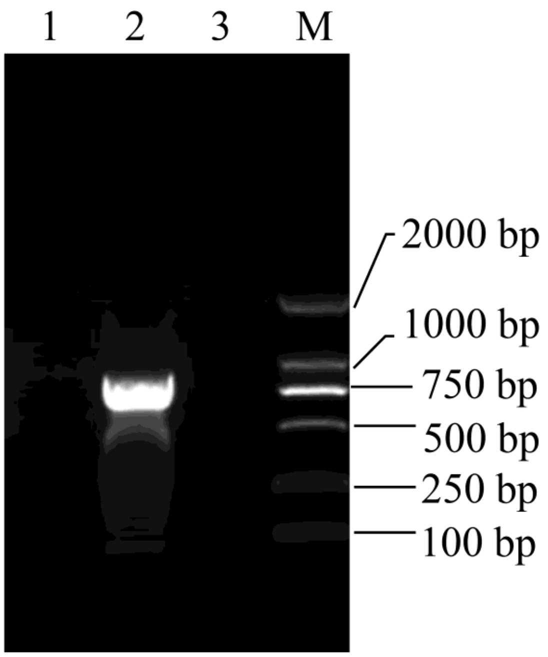

Figure S2. PCR verification of the genome of the red clone

Lane 1: the genome of the red clone; Lane 2: the positive clone containing halogen genes; Lane 3: the negative control; Lane M: DL2000 marker.

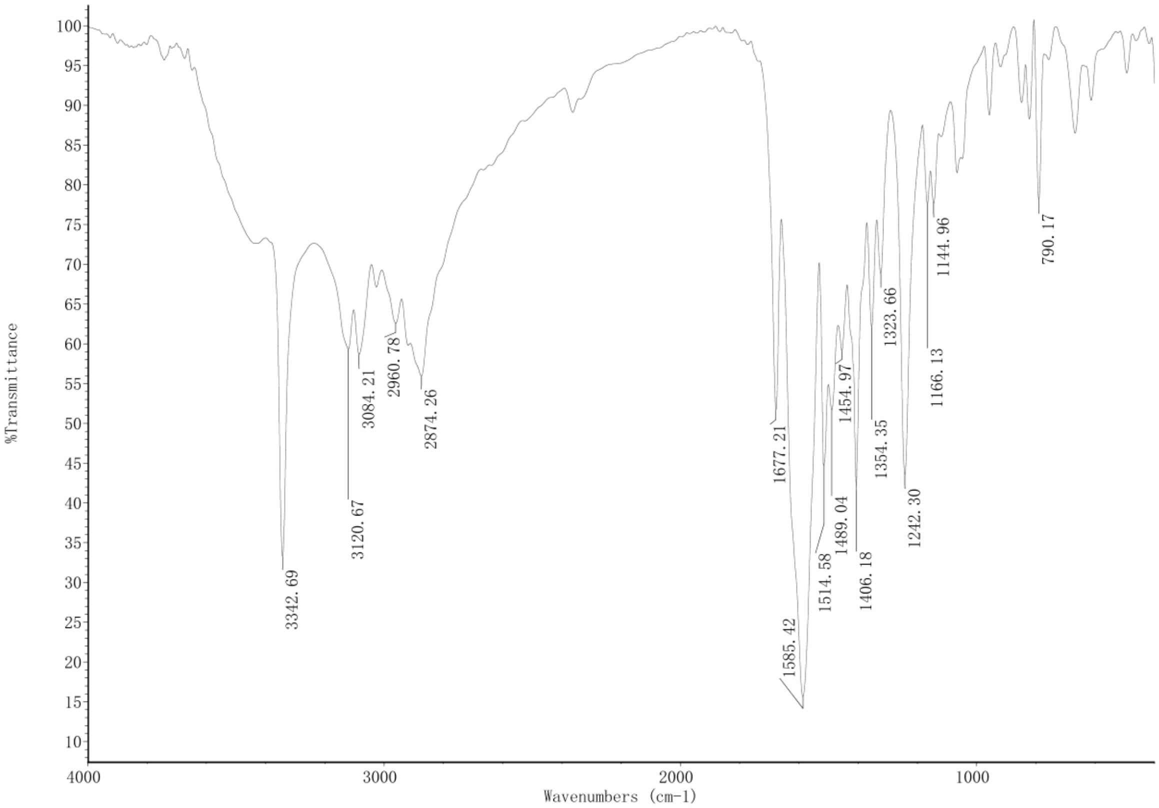

Figure S3. FTIR-spectrum of (**1**)

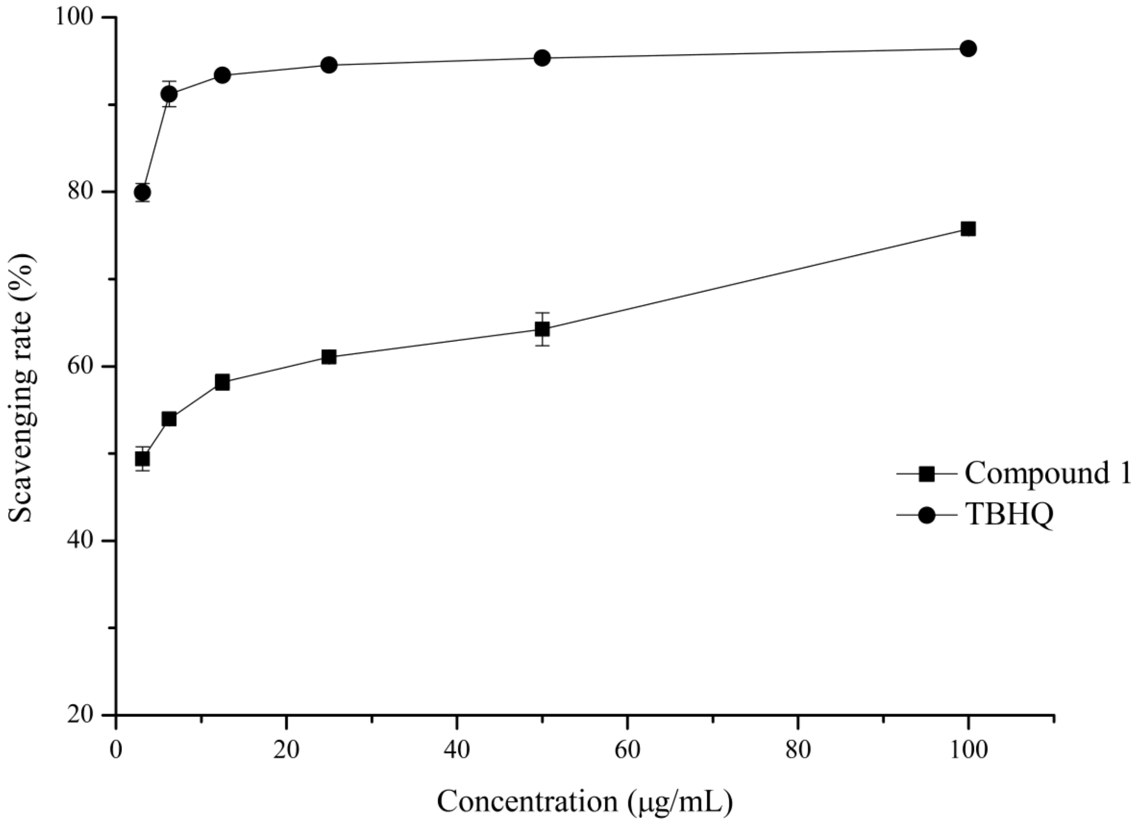

Figure S4. DPPH free radical scavenging activity of (**1**)

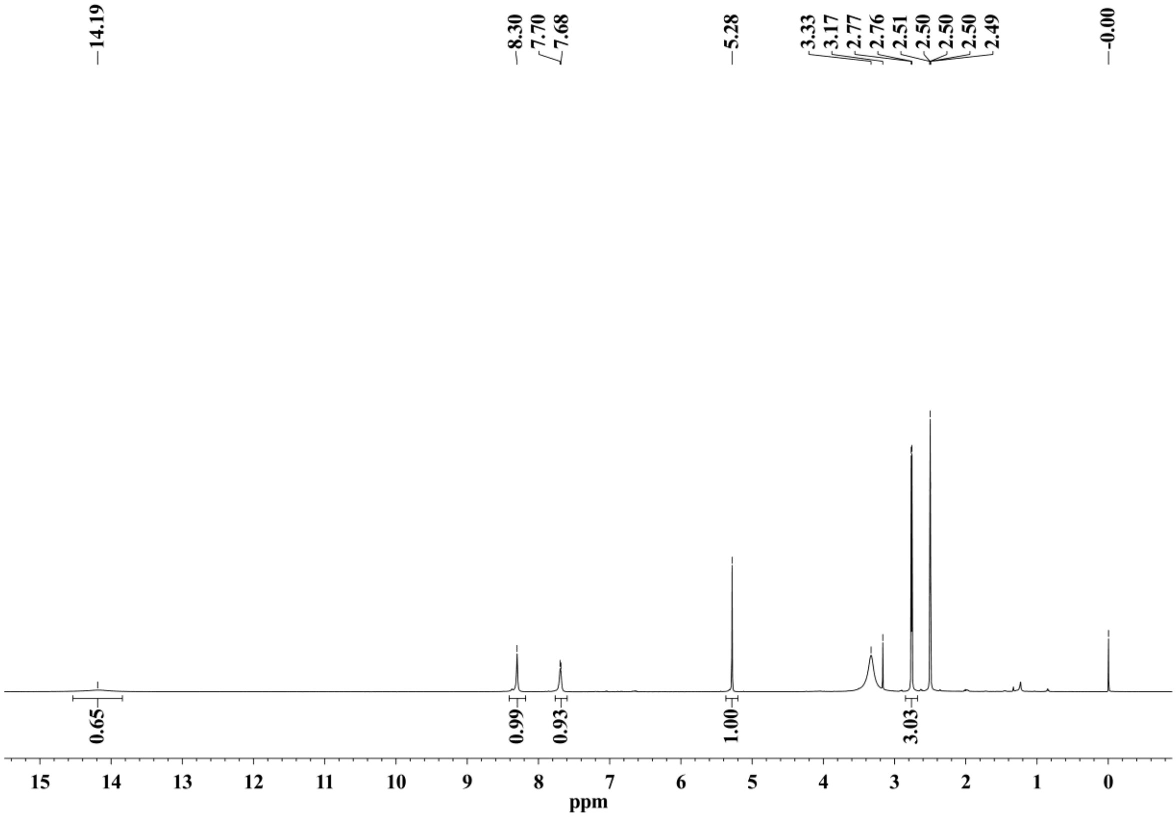

Figure S5. ^{1}H NMR spectrum of (**1**) in DMSO-*d*6

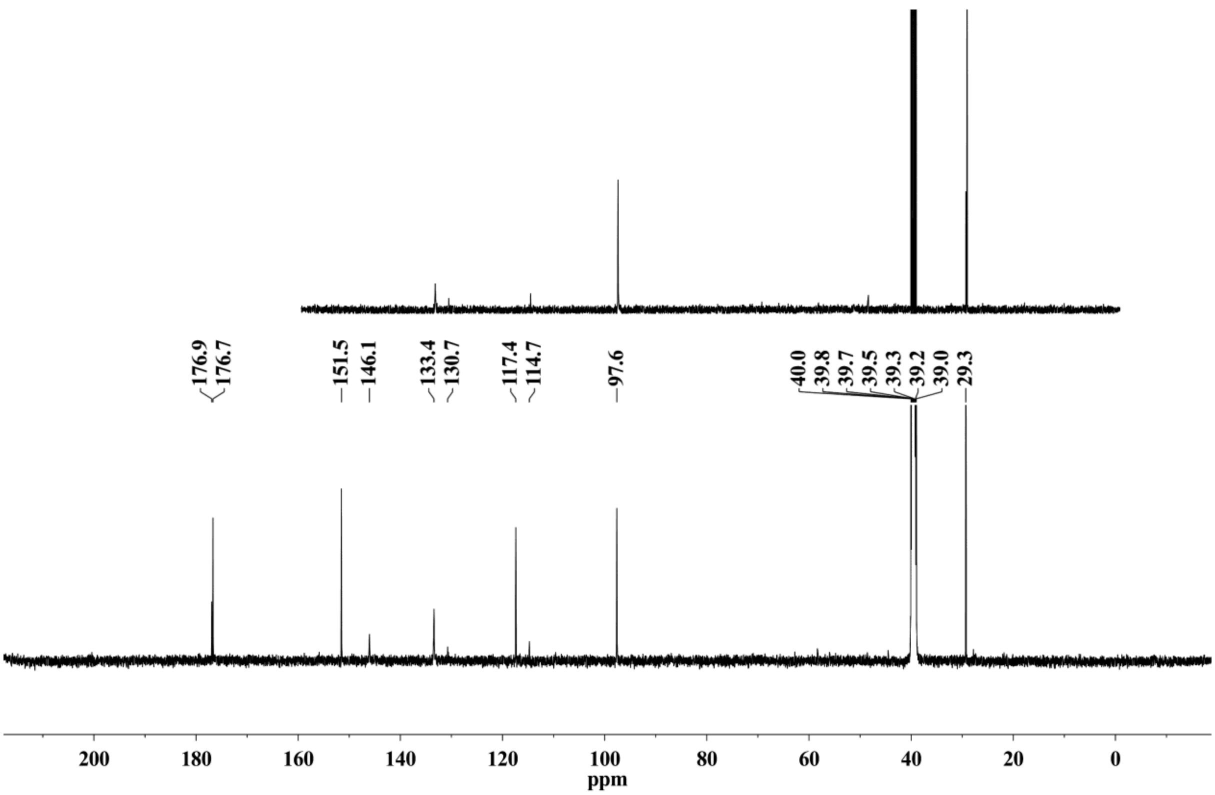

Figure S6**.** DEPT 135 and^{13}C NMR spectrum of (**1**) in DMSO-*d*6

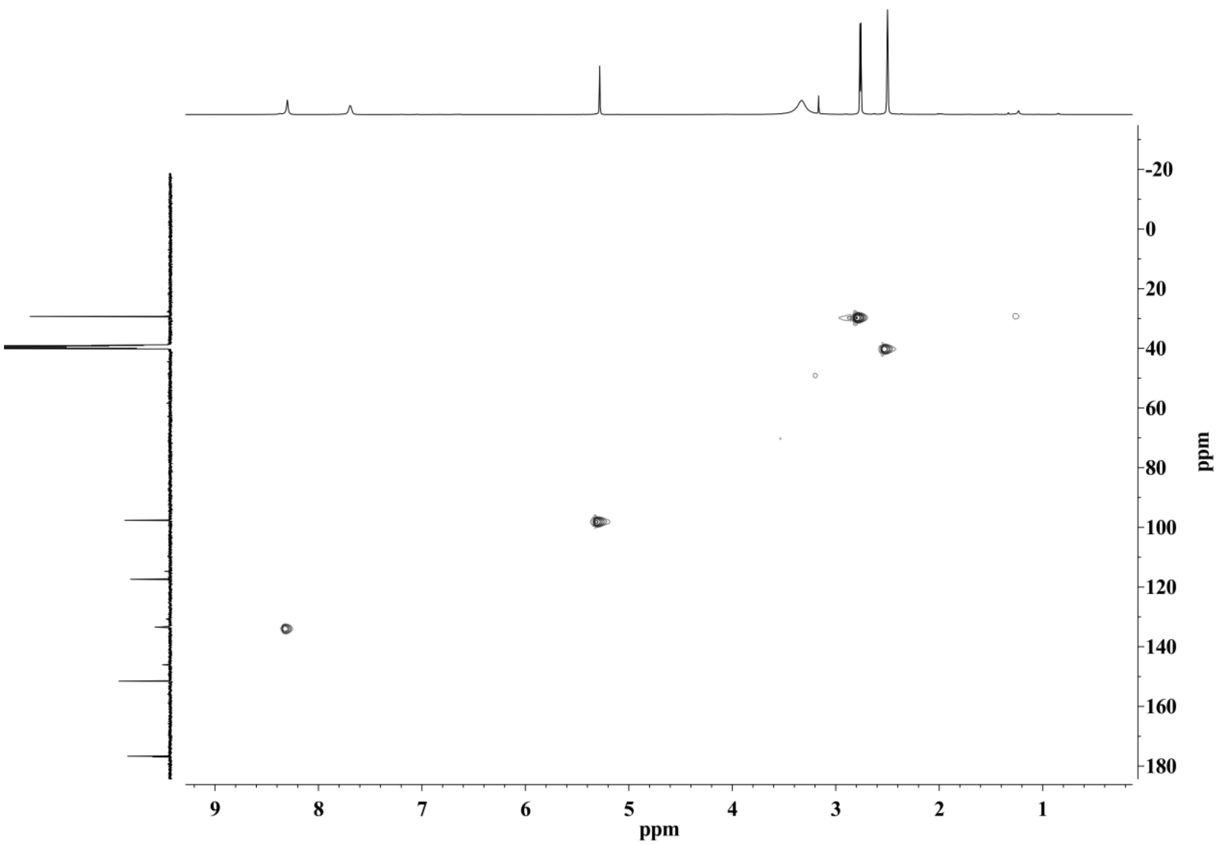

Figure S7**.** HSQC spectrum of (**1**) in DMSO-*d*6

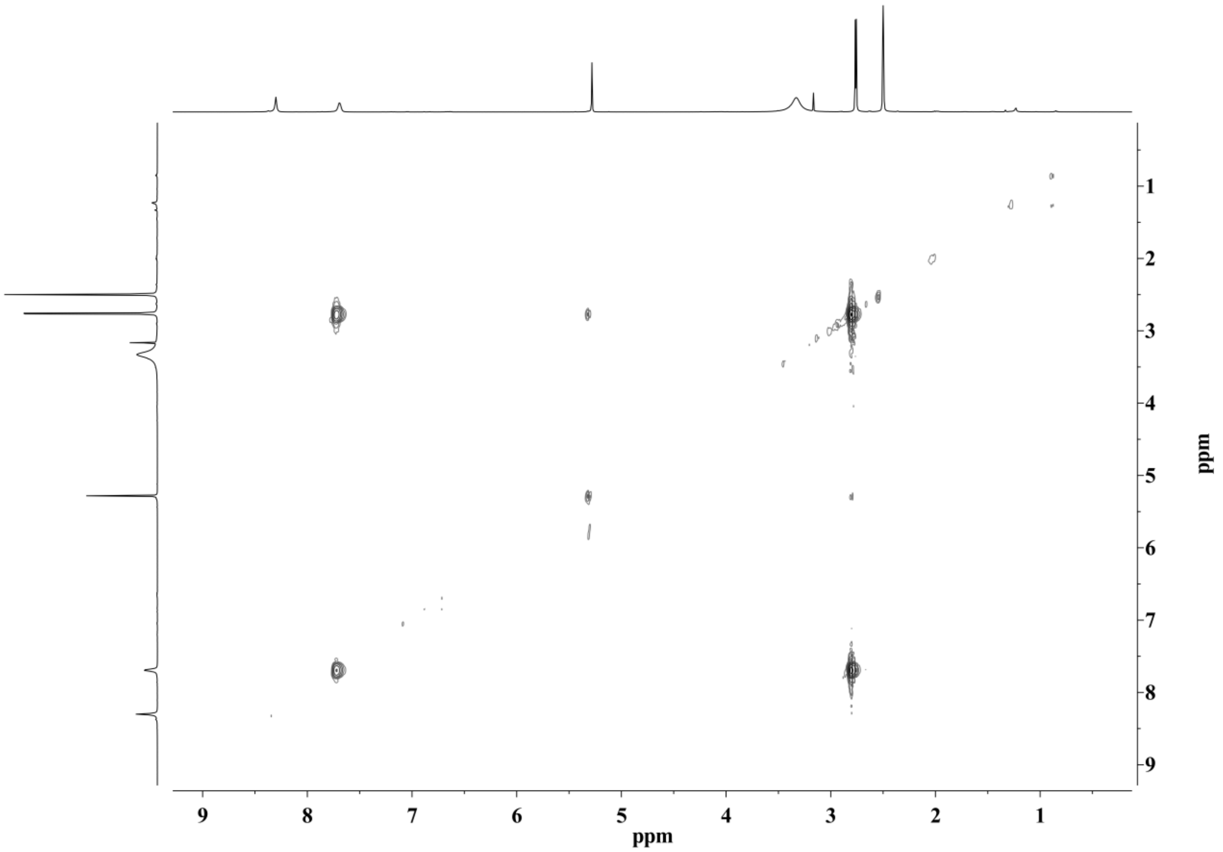

Figure S8**.** COSY spectrum of (**1**) in DMSO-*d*6

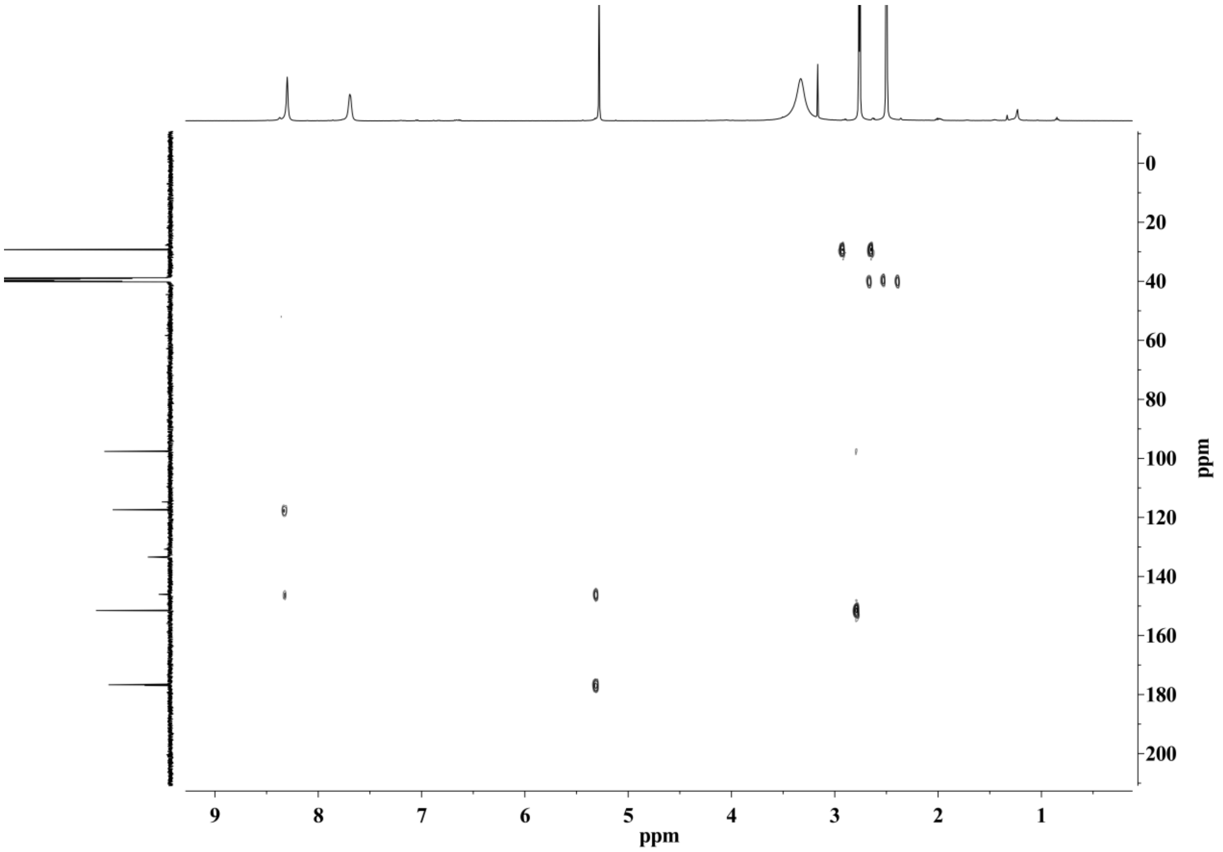

Figure S9**.** HMBC spectrum of (**1**) in DMSO-*d*6

Table S1. Crystal data and structure refinement of (**1**)

Empirical formula	C8 H7 N3 O2	
Formula weight	177.17	
Temperature	296(2) K	
Wavelength	0.71073 Å	
Crystal system	Monoclinic	
Space group	$P2_1/c$	
Unit cell dimensions	a = 4.8856(3) Å	α = 90 °.
	b = 10.0508(7) Å	β = 95.743(2) °.
	c = 16.1190(12) Å	γ = 90 °.
Volume	787.54(9) $Å^3$	
Z	4	
Density (calculated)	1.494 Mg/m^3	
Absorption coefficient	0.112 mm^{-1}	
F(000)	368	
Crystal size	0.220 x 0.180 x 0.150 mm^3	
Theta range for data collection	2.540 to 27.428 °.	
Index ranges	-6<=h<=5, -12<=k<=13, -20<=l<=17	
Reflections collected	8627	
Independent reflections	1786 [R(int) = 0.0257]	
Completeness to theta = 25.242 °	99.4 %	
Absorption correction	Semi-empirical from equivalents	
Max. and min. transmission	0.7456 and 0.6953	
Refinement method	Full-matrix least-squares on F^2	
Data / restraints / parameters	1786 / 0 / 119	
Goodness-of-fit on F^2	1.069	
Final R indices [I>2sigma(I)]	R1 = 0.0422, wR2 = 0.1120	
R indices (all data)	R1 = 0.0533, wR2 = 0.1182	
Extinction coefficient	n/a	
Largest diff. peak and hole	0.287 and -0.227 e.Å-3	

Table S2**.** Bond lengths and angels of (**1**)

**No.**	**Lengths**	**(Å)**	**Angles**	**(°)**
1	C(1)-N(2)	1.3275(17)	N(2)-C(1)-C(6)	112.03(12)
2	C(1)-C(6)	1.4042(19)	N(2)-C(1)-C(2)	125.75(12)
3	C(1)-C(2)	1.476(2)	C(6)-C(1)-C(2)	122.20(12)
4	C(2)-O(1)	1.2374(16)	O(1)-C(2)-C(3)	122.37(13)
5	C(2)-C(3)	1.4239(19)	O(1)-C(2)-C(1)	122.12(12)
6	C(3)-C(4)	1.3730(18)	C(3)-C(2)-C(1)	115.50(11)
7	C(3)-H(3)	0.9300	C(4)-C(3)-C(2)	123.78(13)
8	C(4)-N(1)	1.3263(17)	C(4)-C(3)-H(3)	118.1
9	C(4)-C(5)	1.5145(18)	C(2)-C(3)-H(3)	118.1
10	C(5)-O(2)	1.2179(16)	N(1)-C(4)-C(3)	124.45(12)
11	C(5)-C(6)	1.4431(19)	N(1)-C(4)-C(5)	113.90(11)
12	C(6)-C(7)	1.3778(19)	C(3)-C(4)-C(5)	121.65(12)
13	C(7)-N(3)	1.333(2)	O(2)-C(5)-C(6)	125.15(12)
14	C(7)-H(7)	0.9300	O(2)-C(5)-C(4)	120.25(12)
15	C(8)-N(1)	1.4413(19)	C(6)-C(5)-C(4)	114.58(11)
16	C(8)-H(8A)	0.9600	C(7)-C(6)-C(1)	104.76(12)
17	C(8)-H(8B)	0.9600	C(7)-C(6)-C(5)	132.96(13)
18	C(8)-H(8C)	0.9600	C(1)-C(6)-C(5)	122.27(12)
19	N(1)-H(1)	0.8600	N(3)-C(7)-C(6)	106.16(13)
20	N(2)-N(3)	1.3602(19)	N(3)-C(7)-H(7)	126.9
21	N(3)-H(3A)	0.8600	C(6)-C(7)-H(7)	126.9
22			N(1)-C(8)-H(8A)	109.5
23			N(1)-C(8)-H(8B)	109.5
24			H(8A)-C(8)-H(8B)	109.5
25			N(1)-C(8)-H(8C)	109.5
26			H(8A)-C(8)-H(8C)	109.5
27			H(8B)-C(8)-H(8C)	109.5
29			C(4)-N(1)-C(8)	123.97(12)
30			C(4)-N(1)-H(1)	118.0
31			C(8)-N(1)-H(1)	118.0
32			C(1)-N(2)-N(3)	103.29(12)
34			C(7)-N(3)-N(2)	113.76(12)
35			C(7)-N(3)-H(3A)	123.1
36			N(2)-N(3)-H(3A)	123.1

Table S3. Hydrogen bonds of (**1**)

D-H...A	d(D-H)	d(H...A)	d(D...A)	<(DHA)
N(3)-H(3A)...O(1)#1	0.86	1.90	2.7468(16)	170.0
N(1)-H(1)...O(2)#2	0.86	2.14	2.9134(15)	149.3

Symmetry transformations used to generate equivalent atoms:

#1 -x+2,y+1/2,-z+1/2 #2 -x,-y+1,-z+1
